# Analysis of Peripheral Blood Basophils in Pediatric Systemic Lupus Erythematosus

**DOI:** 10.3390/diagnostics12071701

**Published:** 2022-07-12

**Authors:** Kuanysh Dossybayeva, Yergali Bexeitov, Zaure Mukusheva, Zhaina Almukhamedova, Maykesh Assylbekova, Diyora Abdukhakimova, Marzhan Rakhimzhanova, Dimitri Poddighe

**Affiliations:** 1 Department of Medicine, Nazarbayev University School of Medicine, Nur-Sultan 010000, Kazakhstan; kuanysh.dossybayeva@nu.edu.kz (K.D.); yergali.bexeitov@nu.edu.kz (Y.B.); dabdukhakimova@nu.edu.kz (D.A.); 2 Program of Pediatric Rheumatology, Clinical Academic Department of Pediatrics, National Research Center for Maternal and Child Health, University Medical Center, Nur-Sultan 010000, Kazakhstan; zaure.mukusheva@umc.org.kz (Z.M.); zhaina.almukhamedova@umc.org.kz (Z.A.); maykesh.asylbekova@umc.org.kz (M.A.); 3 Program of Pediatric Endocrinology and Inherited Diseases, Clinical Academic Department of Pediatrics, National Research Center for Maternal and Child Health, University Medical Center, Nur-Sultan 010000, Kazakhstan; marzhan.rahimzhanova@umc.org.kz; 4 Clinical Academic Department of Pediatrics, National Research Center for Maternal and Child Health, University Medical Center, Nur-Sultan 010000, Kazakhstan

**Keywords:** basophils, systemic lupus erythematosus, juvenile idiopathic arthritis, eosinophils, children, fluorescence flow cytometry analysis

## Abstract

Basophils are the least abundant circulating leukocytes, and their immunological role has not yet been completely elucidated. There is evidence supporting their immunomodulatory role in several pathological settings; recently, studies in both experimental models and humans suggested that basophil homeostasis may be altered in systemic lupus erythematosus (SLE). Here, we first assessed circulating basophils in children affected with pediatric SLE (pSLE). In this cross-sectional study, circulating basophils were enumerated by fluorescence-based flow cytometry analysis in children affected with pSLE, in addition to children suffering from juvenile idiopathic arthritis (JIA) or non-inflammatory/non-rheumatic conditions. This study included 52 pediatric patients distributed in these three groups. We observed a statistically significant reduction of peripherally circulating basophils in children with pSLE compared to the other two groups of patients. This preliminary study is consistent with the available studies in adult patients with SLE showing a reduced number of circulating basophils. However, further research is needed to draw final conclusions on basophils’ homeostasis in pSLE, in addition to their correlation with the disease activity and concomitant therapies.

## 1. Introduction

Basophils are the least abundant leukocytes in the bloodstream: indeed, they account for <1% of peripheral blood mononuclear cells (PBMCs). Due to their small number and the difficulties in investigating them, their biological and immunological role has not yet been completely elucidated [1]. However, recent experimental evidence has suggested that basophils are innate immune cells participating in the early phases of the general immunological response, in addition to serving as IgE-driven effector cells after the activation of the adaptive immunity [2]. Indeed, basophils have granules containing a wide range of biologically active substances, including inflammatory mediators (such as vasoactive amines and lipid metabolites), and they can rapidly release several cytokines, including large amounts of IL-4, which can modulate the ongoing immunological response toward a Th2 polarization in several pathological settings, such as respiratory allergy and some parasite infections [3,4,5,6].

Very recently, several authors proposed a potential implication of basophils in autoimmunity, including systemic lupus erythematosus (SLE) [7,8,9]. In this regard, the first hypotheses derived from the observation of basophilia in an experimental murine model developing SLE-like disease (including nephritis), namely the Lyn^−/−^ (which is a Src family protein tyrosine kinase) knock-out murine model; indeed, it exhibits a constitutive Th2-shifted immune response leading to an IgE- and IL-4-dependent nephritis, with glomerular deposition of circulating immune complexes (CICs). Importantly, in these mice the basophil depletion resulted in a reduction of IL-4, IgE levels, and autoreactive antibody production, leading to an improvement of kidney function [10,11]. Similarly, basophil depletion was associated with an improvement of kidney function and survival rate (along with a reduction in circulating autoantibodies) in the murine experimental model MRL/lpr, which is characterized by an autosomal recessive mutation of the lymphoproliferation (lpr) gene (coding Fas antigen); conversely, the adoptive transfer of activated basophils exacerbated the disease progression in these mice [12].

A few human studies explored basophil homeostasis in SLE, but they suggested a perturbation of this cell compartment, as indicated by a reduction of circulating basophils in adult SLE patients [13]. However, no studies investigated basophils in children affected with pediatric SLE (pSLE). Moreover, all these available studies in SLE adults described basophil counts as provided by automated hematology analyzers, whose analytical performance is suboptimal or even inconsistent in comparison with fluorescence-based flow cytometry [14].

In this preliminary research, we aimed to investigate circulating basophils by fluorescence-based flow cytometry analysis in children affected with pSLE.

## 2. Materials and Methods

### 2.1. Study Design and Population

In this cross-sectional study, the main objective was to assess the number of circulating basophils in children diagnosed with pSLE and followed-up at the Rheumatology Program of the Clinical Academic Department of Pediatrics (National Research Center for Maternal and Child Health, University Medical Center) affiliated with the Nazarbayev University School of Medicine.

In addition to pSLE patients, children diagnosed with juvenile idiopathic arthritis (JIA) were included in this study, in order to assess basophils in the setting of a different rheumatic disorder. Moreover, children admitted to the Pediatric Endocrinology Program were also recruited, in order to further compare pSLE study participants with children without rheumatic/systemic inflammatory diseases, defined as non-rheumatic (NR) group. Indeed, the main inclusion criteria in this study were: (i) inpatient admission for diagnosis or regular follow-up of the underlying chronic disorder in the Pediatric Rheumatology Program (in detail, here patients affected with pSLE and JIA were eligible) or in the Endocrinology Program; (ii) being >2 years old and <18 years old; and (iii) permission to collect secondary clinical data and donate a small amount of blood for the study purpose (specific informed consent was signed by the guardians).

### 2.2. Data Collection and Laboratory Analysis

As regards secondary data in rheumatic patients, clinical information (including the parameters to assess the systemic lupus erythematosus disease activity index (SLEDAI) and the 27-joint Juvenile Arthritis Disease Activity Score (JADAS-27)) and laboratory results, including complete blood cell count (CBC) provided by automated analyzer and the main inflammatory parameters, such as C-reactive protein (CRP, n.v. < 10 mg/L) and erythrocyte sedimentation rate (ESR, n.v. < 20 mm/h), were collected. In this regard, each patient underwent these tests (CBC, CRP, and ESR) at the same time when they donated a small blood aliquot to perform basophils analysis by flow cytometry, as described below.

Indeed, the number of circulating basophils was also estimated by flow cytometry and, in detail, by using fluorochrome-conjugated antibodies specific to the high-affinity IgE receptor (FcεRI) and C-C motif chemokine receptor 3 (CCR3). Flow cytometer data acquisition was performed by using FACS MoFlo Astrios EQ (Beckman Coulter, Indianapolis, IN, USA), supported by Summit v6.3.0 software; analysis of acquired data was performed by using FlowJo v10.8.1 software (FlowJo LLC, Ashland, OR, USA). In detail, after lysing red blood cells (ACK lysing buffer, Invitrogen, cat. no. A1049201) of freshly collected blood samples in EDTA, PBMCs were incubated with anti-CD16/CD32 FcR block (Fc Receptor Binding Inhibitor Polyclonal Antibody, eBioscience™, cat. no. 14-9161-73) for 20 min on ice and, eventually (after washing), with anti-FcεRI-FITC (FcεR1 alpha Monoclonal Antibody (AER-37 [CRA1]), FITC, eBioscience™ Invitrogen, cat. no. 11-5899-42) and anti-CCR3-PE (CD193 (CCR3) Monoclonal Antibody eBio5E8-G9-B4, PE, eBioscience™ Invitrogen, cat. no. 12-1939-42) conjugated antibodies for 30 min on ice in a dark environment. Basophils were identified as SSC^low-med^, FSC^low^, FcεRI^+^, and CCR3^+^ PBMCs, according to the current evidence from the medical literature [15,16] and based on our preliminary experiments [17], which showed this population as being CD123^+^ and IL-5R^−^.

### 2.3. Statistical Analysis

The statistical analysis of quantitative variables (expressed as mean ± standard deviation) among the three groups was done by one-way ANOVA (post-test analysis two specific groups, according to Dunn’s multiple comparison test), or unpaired *t*-test with Welch’s correction between two groups. Categorical variables were analyzed through Chi-square test. A *p*-value < 0.05 was considered as statistically significant. The statistical analysis was performed by using Prism 9 for macOS (version 9.3.1, GraphPad software LLC, San Diego, CA, USA).

### 2.4. Ethical Statement

This study has been approved by the Institutional Research Ethics Committee (IREC) of the Nazarbayev University (NU-IREC approval number: 181/23092019, dated on 5 November 2019) and by the Institutional Review Board (IRB) of the University Medical Center (UMC-IRB approval number: 9/2019, dated on 29 November 2019). Informed written consent was obtained by the guardians and, if appropriate, informed written assent form was also obtained by the study participant him/herself.

## 3. Results

### 3.1. Demographics and Main Clinical Characteristics

This study included 52 pediatric patients distributed in three groups: pSLE (n = 17; M/F = 1:16; age: 13.6 ± 2.3 years), JIA (n = 18; M/F = 9:9; age: 13.7 ± 2.5 years), and NR patients (n = 17; M/F = 9:8; age: 12.4 ± 3.2 years). There was no significant difference in age among these three groups; as expected, there was a significant difference (*p* = 0.0385) in gender distribution between the pSLE group and the other two study groups.

As regards patients with pSLE, the SLEDAI was 12.3 ± 11.5; in detail, nine patients had a mild–moderate disease activity (SLEDAI: 0–10; 4.2 ± 3.2) and eight patients showed a high–very high activity (SLEDAI > 10; 21.4 ± 10.6). Ten pSLE patients had a history of nephritis, based on 24-h urinary protein and/or red blood cell casts in urine; however, only two patients underwent renal biopsy, whereas this invasive procedure was not accepted by the guardians of all the remaining patients. As regards the treatment at the time of the study enrollment, all pSLE patients (except one) received hydroxychloroquine (5 mg/kg/day) and 11 were also treated with mofetil-mycophenolate (600 mg/m^2^/12 h), including the one without hydroxychloroquine. Among the six patients without mofetil-mycophenolate, four received methotrexate (15 mg/m^2^/day) and two tocilizumab (10 mg/kg/4 weeks if weight < 30 kg; 8 mg/kg/4 weeks if weight ≥ 30 kg). At the time of the blood sampling, 10 patients were also receiving low-dose steroidal therapy (<9 mg/day on average, corresponding to 0.5 mg/kg/day, in general).

JIA patients were affected with different arthritis subtypes (according to ILAR classifications): oligo-articular (oJIA, n = 6), poly-articular (pJIA, n = 5), enthesis-related arthritis (ERA, n = 2), psoriatic (PsJIA, n = 3), systemic (sJIA, n = 1), and undifferentiated (n = 1). According to JADAS-27, 10 and 8 patients, respectively, showed mild–moderate (score < 6; 3.1 ± 1.1) and high (score ≥ 6; 15.6 ± 8.8) disease activity. As regards the maintenance therapy, at the time of this cross-sectional study, most JIA patients received methotrexate (n = 14; 15 mg/m^2^/day), which was combined with adalimumab in seven cases (20 mg/2 weeks if weight is 10–30 kg; 40 mg/2 weeks if weight ≥ 30 kg) or tocilizumab (10 mg/kg/4 weeks if weight < 30 kg; 8 mg/kg/4 weeks if weight ≥ 30 kg) in one patient. Tocilizumab (12 mg/kg/2 weeks, if weight < 30 kg; 8 mg/kg/2 weeks, if weight ≥ 30 kg) was used in the only patient affected with sJIA. Three patients were treated with etanercept (0.8 mg/kg/1 week).

Patients included in the NR group included the following diagnoses: hypopituitarism (n = 6), Turner syndrome (n = 3), diabetes mellitus type 1 (n = 6), and osteogenesis imperfecta (n = 2).

### 3.2. Hematological and Inflammatory Parameters

CBC, CRP, and ESR are summarized in Table 1. No significant difference was observed for any of the main hematological parameters, except for the absolute eosinophil count. Indeed, pSLE patients showed lower values of eosinophil count (0.15 ± 0.14 × 10^9^/L; *p* = 0.0082) than JIA (0.31 ± 0.38 × 10^9^/L) and NR groups (0.30 ± 0.19 × 10^9^/L). According to the automated analyzer results, no difference in basophil count was observed among these three groups (pSLE: 0.041 ± 0.048 × 10^9^/L; JIA: 0.037 ± 0.011 × 10^9^/L; NR: 0.057 ± 0.018 × 10^9^/L; *p* = ns).

In terms of inflammatory parameters, both CRP and ESR are available for both JIA and pSLE patients, but these tests were not measured in NR children, since they were not necessary for their follow-up, considering the type of diseases included in this group, as described in the previous subsection. Indeed, as explained in the Materials and Methods section, all the study participants were recruited during one periodic admission scheduled to assess the control of their respective underlying chronic disorders.

In terms of inflammatory parameters, most rheumatic patients showed both normal CRP and ESR values: only six JIA patients and seven pSLE patients displayed an increase in one or both parameters. Notably, both CRP and ESR values showed no significant difference between JIA and pSLE patients (CRP: 3.85 ± 6.86 mg/L vs. 4.66 ± 8.20 mg/L, respectively; ESR: 14.0 ± 9.9 mm/h vs. 10.7 ± 12.7 mm/h, respectively).

### 3.3. Peripheral Blood Basophil Count by Fluorescent-Based Flow Cytometry

**Figure 1:**
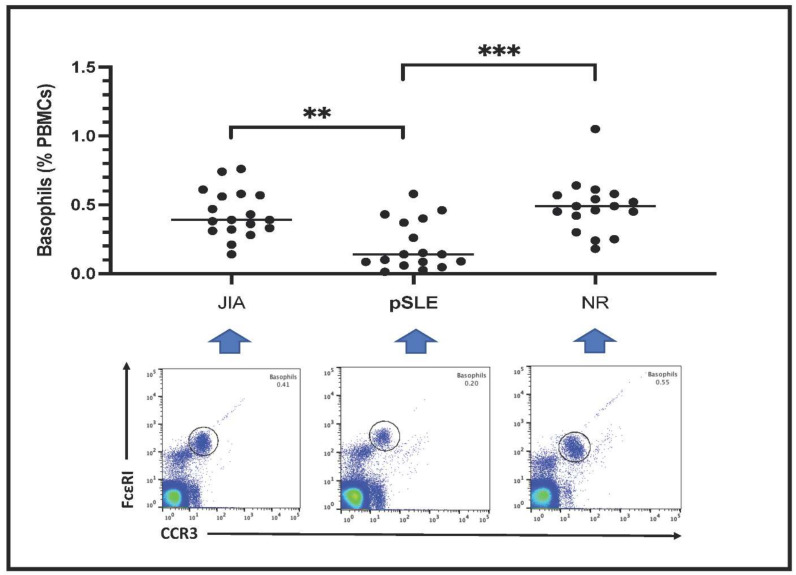
Basophil count in the three groups of patients (**: *p* < 0.01; ***: *p* < 0.001). In the lower part of the figure, an example (one patient) of fluorescence-based flow cytometry plot gating basophils (black circle) is shown. Patients with basophil number close to the mean value of the respective group have been selected as examples.

Circulating basophils are shown as FceRI^+^CCR3^+^ PBMCs in the plots included in Figure 1. Basophil count is expressed as percentage of total PBMCs. Notably, the number of circulating basophils in pSLE patients (0.20 ± 0.18%) was much lower than in children with JIA (0.43 ± 0.17%) or in those affected with non-rheumatic conditions (NR: 0.48 ± 0.20%); indeed, this difference among these groups was highly significant after statistical analysis (*p* = 0.0003).

**Figure 2:**
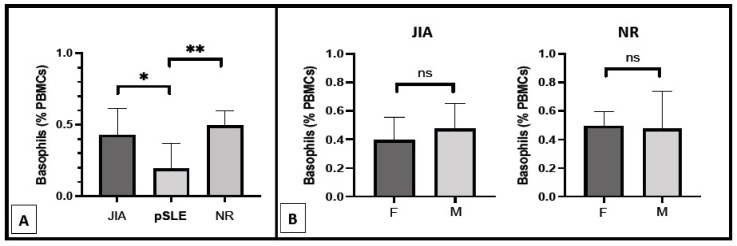
Basophil count in the female patients of the three different groups of diseases ((**A**); *: *p* < 0.05; **: *p* < 0.01) and in pSLE and JIA patients according to gender ((**B**); ns: non-significant; F: female patients; M: male patients).

As described above, all pSLE patients except one were female, and this gender was significantly (*p* = 0.0385) more represented in this group compared to the others (JIA and NR). In order to eliminate any gender-related influence on basophil count, we also analyzed this parameter only in female study participants, as shown in Figure 2A. The inter-groups difference in basophil count remained highly significant (*p* = 0.0015) between pSLE female patients (0.19 ± 0.18%) from one side and female patients belonging to the JIA (0.43 ± 0.19%) and NR (0.49 ± 0.10%) groups on the other side. Accordingly, no difference in basophil number was found between male and female patients of both the JIA and NR groups (Figure 2B).

**Figure 3:**
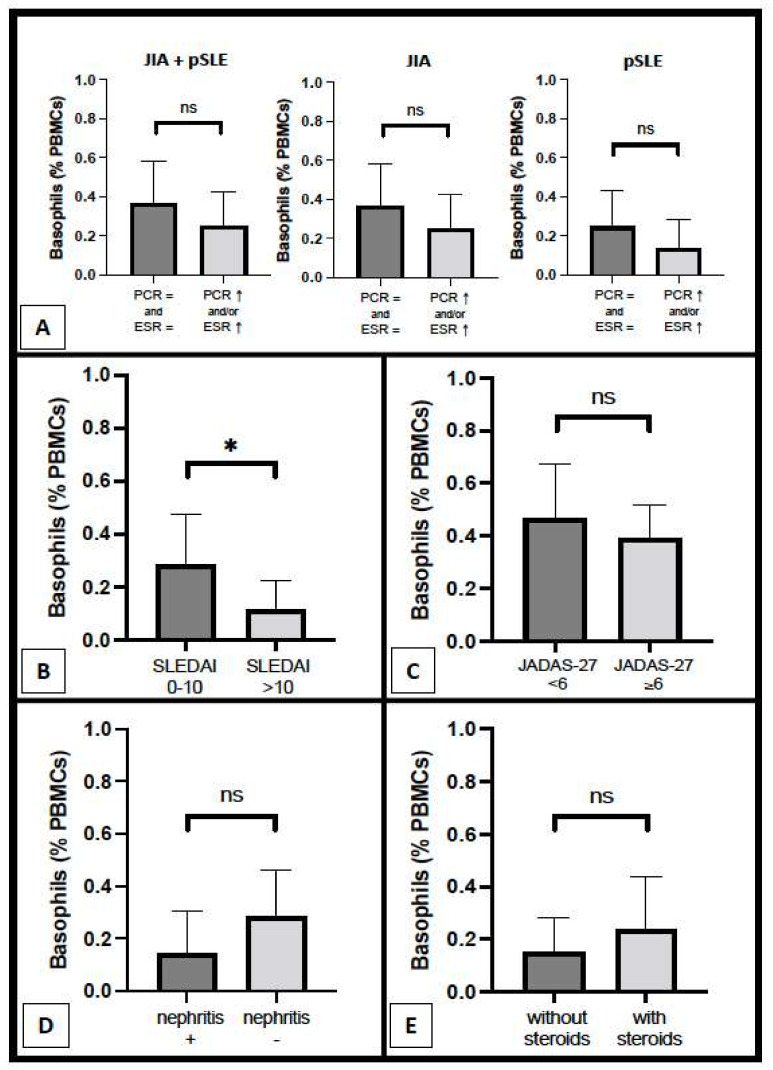
Basophil count according to several parameters, including inflammatory parameters ((**A**) pSLE + JIA combined groups, only JIA group, and only pSLE group), disease activity scores ((**B**) pSLE; (**C**) JIA), and, as regards pSLE patients specifically, renal involvement (**D**) and steroid therapy (**E**) (*: *p* < 0.05; ns: non-significant).

As regards the relation with inflammatory parameters, basophil count was not different between patients with increased CRP and/or ESR and patients with completely normal inflammatory parameters, both in general (0.25 ± 0.18% vs. 0.36 ± 0.22%, respectively; *p* = ns) and inside each disease group (pSLE and JIA), as shown in Figure 3A. Nonetheless, a trend (without any statistical significance) of lowered basophil counts in patients with altered inflammatory parameters can be observed in both pSLE (0.14 ± 0.15% vs. 0.25 ± 0.19%) patients and, to a minor extent, in the JIA (0.38 ± 0.10% vs. 0.46 ± 0.20%) group. Notably, we observed a mildly significant difference in basophil percentage between pSLE patients with mild–moderate disease activity and those with high or very high disease activity based on SLEDAI (0.28 ± 0.19% vs. 0.11 ± 0.11%, respectively; *p* = 0.0451), as shown in Figure 3B; a similar finding was not evident for JIA patients, based on JADAS-27 (Figure 3C). There was no significant difference in basophil number between pSLE patients with and without nephritis (0.14 ± 0.16% vs. 0.28 ± 0.18%, respectively), as shown in Figure 3D; unfortunately, the histopathological examination was available for only two patients (since most of the guardians declined the renal biopsy for their children) and thus, it was not possible to assess the nephritis pattern/degree. Finally, in Figure 3E, we compared basophil count between pSLE children with and without oral steroids (0.24 ± 0.20% vs. 0.15 ± 0.13%, respectively; *p* = ns); however, the sample size was small, and the individual non-steroidal treatments were variable across all these pSLE patients.

## 4. Discussion

In this preliminary study, we investigated circulating basophils in children affected with pSLE and we also compared them with both children affected with another systemic inflammatory rheumatic disease (in detail, JIA) and children diagnosed with non-inflammatory/rheumatic chronic disorders (NR group). To our knowledge, this is the first research assessing blood basophil homeostasis in pSLE.

Notably, we observed a significant reduction of peripherally circulating basophils in pSLE compared with both the JIA and NR groups. This finding is consistent with those few studies that attempted to assess basophil number in adult patients diagnosed with SLE. Liang et al., retrospectively reviewed the clinical records of 213 adult SLE patients and reported a lower circulating basophil number in patients with active disease, suggesting a potential role of this finding as a biological marker for monitoring the clinical course [18]. In a more recent study, the same authors showed that circulating basophil counts were more frequently lower in patients with active lupus nephritis and were negatively correlated with disease activity [19]. However, basophils were counted with an automatic blood cell analyzer, which is not a reliable method to precisely assess such a rare blood cell population [14]. Our study confirms this concern; indeed, the basophil count provided by the automatic analyzer was not able to detect the inter-group difference that was actually shown by basophils analysis through fluorescence-based flow cytometry. Very recently, Jiang Y et al., assessed circulating basophils by multi-parametric flow cytometry in 60 adult patients suffering from SLE and they also described a lower basophil count in these patients (compared to controls) and, in detail, observed a significant difference between SLE patients with and without nephritis [20].

The reduction of circulating basophils may be an intrinsic characteristic of the pSLE phenotype, even though some authors linked this aspect to the disease activity, which would promote their extravasation in the inflamed peripheral tissues [18,19,21,22,23]. Notably, our data showed a mild, but significant, difference between pSLE children with low–moderate and high–very high disease activity, according to SLEDAI, even though we could not demonstrate a similar correlation with the alteration of the inflammatory parameters separately.

However, unlike most JIA patients and NR children, at the time of this cross-sectional study, 10 patients with pSLE were receiving a low dose of steroids. In other pathological settings (such as anaphylaxis and chronic spontaneous urticaria), there is evidence that corticosteroids induce basophil depletion and apoptosis [24,25,26]; of course, the dosage and therapeutic scheme of steroids in these diseases are much higher and different compared to the low steroid regimen administered to pSLE patients in the context of the immunosuppressive maintenance therapy [27]. In the present study, we checked basophil count in pSLE patients on low-dose oral steroid therapy, which was not found to be significantly different from the other pSLE patients. This point was not investigated in the previous and aforementioned studies on adult SLE: Liang et al., reported low-dose prednisone therapy in 35 of their 64 SLE patients with inactive disease, whereas no steroidal therapy was declared for the other 149 SLE patients with active disease (including both patients with nephritis and without this complication) [18]. In their subsequent study, steroids therapy was reported in a similar percentage (around 43–46%) of SLE patients with and without nephritis [19]. In the study by Jiang Y et al., 58 (out of 60) SLE patients were receiving steroids therapy, but this specific aspect has not been explored here either. [20] Although it seems that the reduced number of circulating basophils in SLE patients is present independently from the steroidal therapy, this point should be assessed in patients naïve from any therapy at the time of the pSLE (or adult SLE) diagnosis and, at the moment, there are no specific data in this regard.

Nonetheless, the immunomodulatory role of basophils has emerged from experimental murine models and human studies, and these cells may variably contribute to SLE pathogenesis in cooperation with several elements of both the innate and adaptive immune system [28,29]. Even though their exact role and contribution in SLE development and clinical course has yet to be precisely elucidated, basophils may be able to support a Th2 immunological environment and amplify autoantibody production by B cells (including the production of autoreactive IgE) [12,30]. Moreover, it is interesting to notice the contrast between basophilia (and improvement after basophil depletion) in some murine models of lupus-like disease and the finding of basopenia in human subjects suffering from SLE (in whom the lower levels of basophils seem to correlate with lupus nephritis and negatively with disease activity) [7,9,10,30].

In order to complete the hematological picture, pSLE patients also showed significant reduction in the absolute eosinophil count compared to both JIA and NR children. Most studies providing some information on eosinophil homeostasis in SLE patients are represented by case reports describing its association with some eosinophilic (especially gastro-intestinal) and unusual comorbidities [31,32]. However, we found one study by Yang et al., wherein they analyzed several hematological parameters (including eosinophil count) in different adult autoimmune disorders (including SLE). Notably, in their cohort of 344 adult patients with SLE, these authors described the absolute eosinophil count (0.06 ± 0.15 × 10^9^/L), which was found to be lower (*p* < 0.001) than in healthy controls. Such an eosinophil reduction was not observed for other autoimmune disorders analyzed in this study (namely, rheumatoid arthritis, ankylosing spondylitis, osteoarthritis, and primary Sjogren syndrome). Therefore, our present findings in terms of peripheral eosinophil count seem to be consistent with what was observed in adult pSLE patients by Yang et al. [33]. In this perspective, this additional finding of eosinopenia in pSLE patients and SLE adults [33] might be related to the concomitant basopenia. This is the first time that such a parallelism has been highlighted in SLE, to our knowledge. Both eosinophils and basophils may be similarly subjected to extravasation into peripheral tissues. The correlation of lower blood basophil counts in pSLE children with higher disease activity and the same trend according to the elevation of inflammatory parameters and/or the presence of renal involvement (that we noticed in this study too) may be consistent with this hypothesis.

However, several limitations affected this preliminary study, including the small sample size and the fact that study participants were not naïve in terms of pharmacological therapy and, in detail, most pSLE patients were also receiving low-dose oral steroids at the time of the blood collection. Moreover, because of the hospital admission limited to children suffering from chronic disorders, we could not include completely healthy children as a non-rheumatic control group. Therefore, these preliminary findings in pSLE definitely need further confirmation from larger and standardized clinical studies, possibly including both rheumatic patients at the time of the first diagnosis (then, without any immunomodulating and/or steroid therapy) and healthy controls.

## 5. Conclusions

Our preliminary study first assessed the basophil count in the peripheral blood of children affected with pSLE by multicolor flow cytometry analysis, which was found to be lower than in patients with JIA or those suffering from non-inflammatory disorders. Eosinophil count was also reduced in these pSLE patients. Even though our observations are consistent with the previous studies on adult patients with SLE in terms of the lower number of circulating basophils, these cells should be assessed in SLE patients at the diagnosis, before starting any steroidal and non-steroidal immunosuppressive therapy. Therefore, further research is needed to draw final conclusions on the basophils’ homeostasis in pSLE, in addition to their correlation with the disease activity and concomitant therapies.

## Figures and Tables

**Table 1:** Complete and differential blood cell count and inflammatory parameters in the study population.

Hematological Parameters	JIA	pSLE	NR
			
**HGB** (g/L)	129.9 ± 13.9	125.7 ± 18.1	130.8 ± 10.7
			
**PLT** (10^9^/L)	292 ± 81	288 ± 107	356 ± 84
			
**WBCs** (10^9^/L)	7.7 ± 2.4	8.2 ± 4.7	7.2 ± 1.5
			
**Lymphocytes** (10^9^/L)	3.1 ± 0.7	2.5 ± 1.1	3.3 ± 1.0
			
**Neutrophils** (10^9^/L)	3.6 ± 2.1	5.3 ± 4.1	2.9 ± 1.0
			
**Monocytes** (10^9^/L)	0.68 ± 0.23	0.72 ± 0.35	0.57 ± 0.11
			
**Eosinophils** (10^9^/L)	0.30 ± 0.39	0.15 ± 0.14 *	0.30 ± 0.19
			
**Basophils** (10^9^/L)	0.037 ± 0.011	0.041 ± 0.048	0.057 ± 0.018
			
**CRP** (mg/L)	3.85 ± 6.86	4.66 ± 8.20	n/a
			
**ESR** (mm/h)	14.0 ± 9.9	10.7 ± 12.7	n/a

Abbreviations: HGB: hemoglobin; WBCs: white blood cells; PLT: platelets; ESR: erythrocyte sedimentation rate; CRP: C-reactive protein; n/a: not available. * Statistically significant difference from the other two groups.

## Data Availability

The data presented in this study are available upon request from the corresponding author. The data are not publicly available due to confidentiality reasons.
